# The Interfacial Interaction of Functional Liquid Polyisoprene Rubber in SSBR/Silica Composite

**DOI:** 10.3390/polym18121416

**Published:** 2026-06-06

**Authors:** Ji Ma, Zhixuan Yan, Dandan Liu, Guangye Liu, Naixiu Ding, Lixia He

**Affiliations:** 1 College of Polymer Scienceand Engineering, Qingdao University of Science and Technology, Qingdao 266042, China; 02630@qust.edu.cn; 2 Neijiang Economic Cooperation Bureau, Neijiang 641100, China; 4019020062@mails.qust.edu.cn; 3 Engineering Research Center of High-Performance Polymer and Molding Technology, Ministry of Education, Qingdao University of Science and Technology, Qingdao 266042, China; liudandan0305@126.com (D.L.); 00515@qust.edu.cn (G.L.); 02095@qust.edu.cn (N.D.)

**Keywords:** liquid polyisoprene rubber, anionic polymerization, end-functionalized, dispersant

## Abstract

Silica dispersion in rubber matrices remains a critical issue due to the polarity mismatch between silica and the rubber phase. This study aimed to synthesize functionalized liquid polyisoprene rubber (F-LIR) and evaluate its role in improving the interfacial interaction between silica and solution styrene–butadiene rubber (SSBR). F-LIR was synthesized by introducing an alkoxysilane-containing functionalizing agent at the termination stage of anionic polymerization. Fourier transform infrared spectroscopy (FT-IR) and proton nuclear magnetic resonance spectroscopy (^1^H-NMR) were used to confirm the successful introduction of silyl groups at the chain ends of liquid polyisoprene. The optimal loading of F-LIR in SSBR was evaluated through bound rubber content, dynamic mechanical analysis, and mechanical performance testing. The results demonstrated that F-LIR improved the tensile strength, modulus at 300% elongation, and bound rubber content of SSBR composites. These enhancements are attributed to the reaction between the silyl groups of F-LIR and surface hydroxyl groups of silica, together with the co-crosslinking interaction between F-LIR and SSBR. The composites containing 4 phr F-LIR exhibited the best overall balance of properties. This study provides a novel method for synthesizing F-LIR, which bridges silica and the rubber matrix by enhanced filler–rubber interactions at the filler–rubber interface.

## 1. Introduction

With the gradual rise in the global temperature and the non-renewable characteristics of oil, the eco-friendly and sustainable materials should be paid more attention [1,2,3,4]. As one of the petrochemical products, tires play a significant role in the automobile field. Many researchers have focused on how to improve the sustainability and environmental friendliness of tire products as a research topic [5,6].

In the rubber processing process, plasticizers are widely used because it will increase the distance between the rubber matrix molecular chain and reduce the Mooney viscosity and the cost of rubber products [7,8,9]. With the continuous development of polymer and rubber industries, the demand for plasticizers has steadily increased in recent years [10]. However, traditional plasticizers such as aromatic oil, naphthenic oil and paraffin oil are easy to migrate to the surface of vulcanizates, which will cause size shrinkage of rubber products, and volatile gas will lead to serious environmental pollution [11,12,13,14]. As early as June 2007, the REACH regulation [15] of EU (European Union) has come into effect, which clearly stipulates that rubber products exported to the European Union are prohibited from containing aromatic hydrocarbons. Therefore, much attention has been focused on reactive plasticizers in recent years.

Compared with conventional plasticizers, liquid polyisoprene rubber (LIP) has emerged as a promising reactive plasticizer due to its low volatility, co-crosslinking capability with rubber matrices, and potential to improve filler dispersion [11,16]. Previous studies demonstrated that LIR-containing rubber composites exhibit improved dimensional stability, thermal aging resistance, and wet skid performance compared with those containing traditional oils [11,16]. More importantly, the unsaturated double bonds in LIR can participate in vulcanization reactions, thereby reducing plasticizer migration and improving the durability of rubber products. Nevertheless, conventional LIR primarily functions as a processing aid and lacks the capability to establish strong interfacial interactions with inorganic fillers.

Compared with carbon black in rubber-reinforced system, silica, as a non-petroleum-based reinforced material, has the characteristics of low rolling resistance, good skid resistance and environmental friendliness. Therefore, silica is often used in styrene butadiene rubber filling system for the preparation of high performance, energy saving “green tire” [17,18]. However, the abundant hydroxyl groups on the silica surface impart strong polarity, leading to severe incompatibility with nonpolar elastomers such as SSBR. This polarity mismatch generally results in strong filler–filler interactions, silica agglomeration, and insufficient filler–rubber interfacial adhesion, thereby deteriorating the mechanical and dynamic properties of vulcanizates [19,20,21,22]. Therefore, improving silica dispersion and strengthening filler–rubber interfacial interactions remain major challenges in silica-reinforced rubber systems.

To address these issues, several strategies have been proposed. Silane coupling agents, particularly bis(triethoxysilylpropyl)tetrasulfide (TESPT), are commonly employed to reduce silica agglomeration and improve filler–rubber compatibility through silanization reactions [23,24,25,26,27]. In addition, functional polymers containing polar groups capable of hydrogen bonding have been developed to improve silica dispersion. Weng et al. [28] have successfully synthesized a polymer with imidazole onto the main chain of SSBR via thio-ene click reaction, which promote the dispersion of silica through hydrogen bonding. Qiao et al. [29,30] investigated whether the epoxy group-functionalized polymer (PDBIIG) via free-radical redox emulsion polymerization promoted the dispersion of silica. The results indicate that the ring-opening reaction and the effect of hydrogen bonding between the hydroxyl groups on the surface of silica and the epoxy groups of PDBIIG played a significant role in strengthening the interfacial interaction between silica and rubber. Introducing alkoxysilane groups into polymer chains has also been demonstrated to be an effective strategy for improving silica dispersion. Liu et al. [31] and Gao et al. [32] have successfully synthesized a multifunctional solution polymerization styrene butadiene rubber (SSBR) by connecting the end of SSBR and the main chain of SSBR with alkoxysilane groups respectively. The former polymer via the end-group modify method can not only significantly reduce the free ends of macromolecular chains but also form more chemical bonds between silica and rubber matrices. The latter polymer was linked to the alkoxysilane groups on the SSBR backbone by thio-ene click reaction, which would attach more alkoxysilane groups to show excellent performance such as rolling resistance, wet skid resistance and tensile strength. However, a chemical combination of silane and reactive plasticizer is applied in styrene butadiene rubber, which has rarely been discussed.

In this study, a multifunctional end-functionalized liquid polyisoprene rubber (F-LIR)-containing alkoxysilane groups was successfully synthesized through end-group modification during anionic polymerization. Unlike conventional plasticizers, the designed F-LIR simultaneously performs three functions: plasticization, filler dispersion, and co-crosslinking. Specifically, the low molecular weight of F-LIR facilitates chain mobility and improves processability, while the unsaturated backbone enables co-vulcanization with the SSBR matrix. More importantly, the alkoxysilane groups introduced at the chain end can react with silanol groups on the silica surface, thereby strengthening filler–rubber interfacial interactions and suppressing silica aggregation. As a result, F-LIR acts as a multifunctional interfacial bridge between silica and the rubber matrix. The chemical structure and functionalization efficiency of F-LIR were characterized using Fourier transform infrared spectroscopy (FT-IR) and proton nuclear magnetic resonance spectroscopy (^1^H-NMR). Furthermore, the effects of F-LIR on curing behavior, filler dispersion, bound rubber content, crosslink density, mechanical properties, and dynamic mechanical performance of SSBR/silica composites were systematically investigated to determine the optimal loading level and clarify the underlying reinforcement mechanism.

## 2. Materials and Methods

### 2.1. Raw Materials and Equipment

Isoprene (gas chromatographic purity) and n-hexane (analytical grades), purchased from Shanghai Macklin Biochemical Co., Ltd. (Shanghai, China), were dried over calcium hydride and distilled in dry nitrogen to create an ambient free of water and oxygen. n-butyllithium (n-BuLi) and 4-vinyl pyridine, supplied by Shanghai Aladdin Bio-Chem Technology Co., Ltd. (Shanghai, China), were used as the initiator and color agent, respectively, in the anionic polymerization. The 3-Glycidyloxypropyltrimethoxysilane (KH-560), also purchased from Shanghai Aladdin Bio-Chem Technology Co., Ltd., plays a role of end-capping reagent at the end of the polymerization reaction. Other raw materials commonly known to be used in tires were used: Commercial solution polymerized styrene–butadiene rubber (SSBR, 2525) was produced by the Jiangsu Aitong international trading Co., Ltd. China (Jiangyin, China). Silica was used as the reinforcing filler. Zinc oxide (ZnO), stearic acid, poly(1,2-dihydro-2,2,4-trimethyl-quinoline) (RD), sulfur, N-tert-butylbenzothiazole-2-sulphenamide (TBBS), 1,3-Diphenylguanidine were industry grade and used as additives in the curing package.

### 2.2. Synthesis of Functionalized Liquid Polyisoprene

Functionalized liquid polyisoprene (F-LIR), synthesized in our laboratory, was conducted as described subsequently. Because the anion active species is easily inactivated, the whole polymerization system must be strictly water-free and oxygen-free. Precisely under such conditions, n-hexane and isoprene must be refluxed with calcium hydride, distilled with dry nitrogen, and immersed in molecular sieves (5 Å) before being used. Functionalization agents must be further purified.

**Figure 1:**
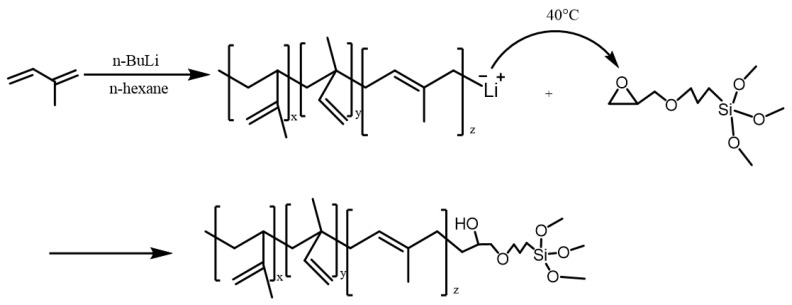
Synthetic mechanism of functionalized liquid polyisoprene.

After all the reagents have been pretreated, n-hexane and isoprene with a solubility of 20% monomer (e.g., 20 g of isoprene in 80 g of n-hexane) were imported into the polymeric flask which has been roasted three times with a hot air gun and washed with n-BuLi solution beforehand. The polymerization was initiated by n-BuLi, which had been calculated based on the number of contaminants in the whole system and the expected molecular weight of the liquid polyisoprene. The mixed solution was stirred for 4 h under 40 °C. At the end of the polyreaction, functionalization agents were added 1.5 times as much as the anion active species. Ethanol, used as the coagulant agent of the F-LIR and the detergent for redundant functionalization agents, was added to the mixture. To remove the excess KH-560 that is not connected at the end of LIR, the F-LIR, flocculated by absolute ethyl alcohol, dissolves in n-hexane. With the continuous-flocculation process, the KH-560 which did not participate in the capping reaction was effectively removed. The polymer was dried to a constant weight in a vacuum oven at 50 °C. The specific reaction mechanism of F-LIR is shown in Figure 1.

### 2.3. Preparation of SiO_2_-g-F-LIR Masterbatch

Silica with more hydroxyl groups on the surface has higher polarity, and it has the characteristics of small particle size and large specific surface area, whereas a large proportion of rubber are nonpolar, and styrene butadiene rubber is no exception. However, the highly polar surface of silica makes homogeneous dispersion in nonpolar rubber matrices difficult. It is a good idea for removing the hydroxyl groups on the silica surface where the silica was combined with F-LIR.

**Figure 2:**
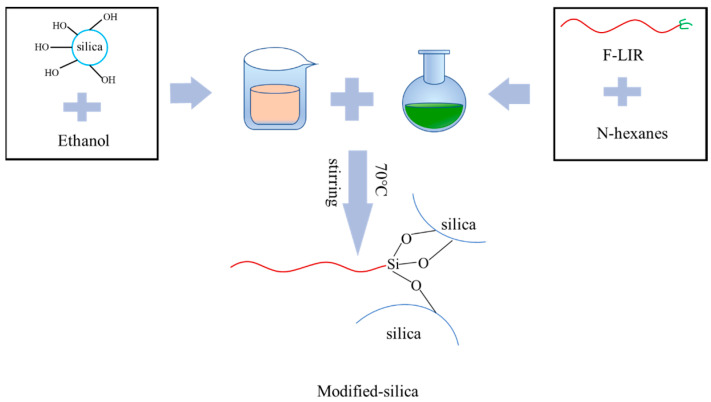
Schematic illustration of the preparation of SiO_2_-grafted F-LIR modified silica masterbatch.

First, the corresponding mass of F-LIR was added into n-hexane to form the mixed solution with a concentration of 20%. Waiting for the F-LIR to dissolve completely, the mixture of silica combined with ethanol was added. All the agents were added into the glass flask, followed by heating the mixed solution up to 70 °C and stirring it for 4 h. Then, after the mixture was dried in the vent light window for 2 days, the SiO_2_-g-F-LIR mixture was dried to a constant weight in a vacuum oven at 50 °C. The schematic diagram is shown in Figure 2.

### 2.4. Blending and Curing

The recipe for all the compounds is shown in Table 1 and the filling factor is 0.75. The compounds were prepared through a two-step mixing procedure by using a Haake Polylab OS and a two-roll mill from Thermo Fisher Scientific (Waltham, MA, USA) and Shanghai Shuangyi Rubber and Plastic Machinery Co., Ltd. (Shanghai, China), respectively.

In the first step, the SSBR was added into a Haake internal mixer which used Banbury rotor and were mixed at 60 rpm. After about 1.30 min, ZnO, stearic acid, and antioxidant RD were mixed with rubber matrix. Once the torque was in equilibrium, premixed SiO_2_-g-F-LIR admixture and remaining silica were added. After mixing for about 2 min, the rubber compound was discharged when the temperature in the chamber exceeded 120 °C. During the second step, sulfur and accelerators were mixed in a two-roll mill at a roll temperature of 50 °C. When all the reagents are mixed evenly, the compounds were sheeted about 2 mm thickness. Rubber products must be vulcanized to be useful. After blending, the compounds were cured in different test specimen molds with a temperature of 160 °C for the optimum curing time t_90_. The pressure of hot press was 10 MPa.

### 2.5. Methods

The microstructure and the end-functionalization efficiency of F-LIR were characterized by FT-IR, ^1^H-NMR, gel permeation chromatography (GPC). The application performance of vulcanized film was verified by tensile strength, crosslinking density, dynamic mechanical analysis (DMA) and other test items.

#### 2.5.1. FT-IR

F-LIR, dried in the vacuum oven, was evenly smeared on the KBr sheet. It was observed by Vertex Fourier transform infrared spectrometer (Bruker Corp., Berlin, Germany) under the range of wavelength of 4000–400 cm^−1^ at a resolution of 4 cm^−1^ with 32 scans.

#### 2.5.2. ^1^H-NMR

The end-functionalization efficiency of F-LIR was analyzed by ^1^H nuclear magnetic resonance (Avance 500 MHz, Bruker Corp., Fällanden, Switzerland). About 20 mg of F-LIR was dissolved in CDCl_3_ containing 0.03% TMS at room temperature. The samples were tested at room temperature using TMS as internal standard. The calculation formula of the end-functionalization efficiency was as follows:

(1) $$E=Mn\times M_{\left(\text{PI-1},4\%\right)}\times 1\times S_{\left({\text{-OCH}}_{3}\right)}/68\times 9\times S_{\left(\text{PI-1},4\right)}$$

(2) $$M_{\left(\text{PI-1},4\%\right)}=S_{\left(\text{PI-1},4\right)}/(S_{\left(\text{PI-1},4\right)}+S_{\left(\text{PI-3},4\right)})$$

where *E* represents the end-functionalization efficiency of F-LIR, *M*n is the number-average molecular weight of F-LIR, M_(PI-1,4%)_ is the molar percentage of 1,4-structure in F-LIR, *S*_(-OCH3)_ represents the peak area of characteristic peak of alkoxysilane groups connected at the end of LIR, *S*_(PI-1,4)_ and *S*_(PI-3,4)_ represents the peak area of 1,4-structure and 3,4-structure in F-LIR, respectively.

#### 2.5.3. GPC

The number-average molecular weight (Mn) and the molecular weight distribution (PDI) were measured at 40 °C by GPC (Waters 150C, Milford, MA, USA). An amount of 50 mg of LIR or F-LIR sample was dissolved in 10 mL of tetrahydrofuran to prepare a solution with a concentration of 5 mg·mL^–1^. Tetrahydrofuran was used as the mobile phase at a flow rate of 1 mL·min^–1^. Polystyrene standards were adopted for calibration.

#### 2.5.4. Curing Characteristics

The curing behavior of the compounds were investigated at 160 °C with a test time of 50 min by using a rubber process analyzer (RPA) (RPA 2000, Alpha Corp., Sterling, VA, USA). Various curing parameters, such as the scorch time, t_10_ which was related to the safety of rubber processing; optimal curing time, t_90_, was the time to complete rubber processing; cure rate; the highest maximum torque values (MH) and the lowest minimum torque values (ML) were acquired from the curing curves.

#### 2.5.5. Crosslinking Density

Crosslinking density values [33] were attained by swelling test. About 1 g of vulcanizates were soaked into toluene at room temperature for 72 h; the fresh toluene was replaced every 36 h. Finally, after swelling vulcanizates reach equilibrium, a filter paper was used to wipe off the excess toluene on its surface immediately and were weighed accurately. According to the Flory–Rehner theory [34], the crosslinking density values can be calculated as follows:

(3) Xc=−v2+χv22+ln(1−v2)v1(v213−v22)

where X_c_ represents the crosslink density (g·cm^−3^) of vulcanizates, *v*_2_ is the volume fraction obtained by Equation (4), χ expressed Flory–Huggin’s parameters, which is the interaction parameter between polymer and solvent, *v*_1_ represents the molar volume of solvent (toluene = 106.29 cm^3^·mol^−1^). The Flory–Huggins parameter for SBR/toluene system is 0.446.

(4) $$v_{2}=\frac{m_{2}/\rho_{2}}{\left(m_{2}/\rho_{2})+(m_{1}/\rho_{1}\right)},$$

where m_1_ (g) and m_2_ (g) are the solvent weight in the equilibrium state of swelling and the unswollen specimen mass respectively, and the ρ_1_ (for toluene = 0.867 g·cm^−3^) and ρ_2_ represent the solvent density and the specimen density respectively.

#### 2.5.6. Determination of Bound Rubber Content

The bound rubber content was able to reflect the dispersion of filler in unvulcanized rubber blends. Firstly, about 0.5 g rubber compounds which were cut into small particles were enclosed in a 300-mesh stainless steel meshwork. Secondly, it was immersed in toluene at room temperature for 3 days and then in acetone for 1 day. Finally, it was dried to constant weight in a vacuum oven at 50 °C and weighed accurately. The bound rubber content was calculated by the following formula [35,36]:

(5) $$R_{b}(\%)=100\times \left.\left\{W_{fg}-W_{t}\left[m_{f}/\left(m_{f}+m_{r}\right)\right]\right.\right\}/\left\{W_{t}\left[m_{r}/\left(m_{f}+m_{r}\right)\right]\right\}$$

where R_b_ is the bound rubber content of compounds, W*_fg_* the weight of the sample after soaking and drying, W*_t_* the weight of the specimen, *m_f_* and *m_r_* the fraction of filler and rubber in the compound respectively.

#### 2.5.7. Determination of Physical Mechanical Properties

The tensile testing was analyzed by using tensile tester (GOTECH Testing Machines Co., Ltd., Taichung City, Taiwan) in accordance with ASTMD412-16(2021) [37] at room temperature. The measurements were carried out at a crosshead speed of 500 mm/min. The tensile strength, elongation at break, stresses at 100% and 300% strain were obtained from the stress–strain curve. The hardness of vulcanizates was determined by using a Durometer type A.

#### 2.5.8. Determination of Dynamic Mechanical Properties

The dynamic mechanical property was determined in a tensile mode by using Dynamic Mechanical Thermal Analyzer (DMTA, Netsch Gabo Corp., Ahlden, Germany). For the temperature sweep, the temperature range was from −90 °C to 120 °C with a heating rate of 3 °C/min. and the frequency was 10 Hz. For the strain sweep, the strain range was from 0.1% to 20% at 10 Hz and the temperature was 40 °C. The elastic modulus, loss modulus and tanδ were acquired through these two kinds of sweep.

## 3. Results

### 3.1. Synthesis and Characterization of the F-LIR

The anion polymerization method was adopted in the synthesis of polymer process. The critical step of the reaction was to add KH-560 agents to the end of the anion polymerization to inactive the anion active species at 40 °C for 1 h, which would determine whether the alkoxysilane groups were successfully attached to the end of LIR. The successful end-functionalization of LIR and the functionalization efficiency were characterized by FT-IR and ^1^H-NMR.

**Figure 3:**
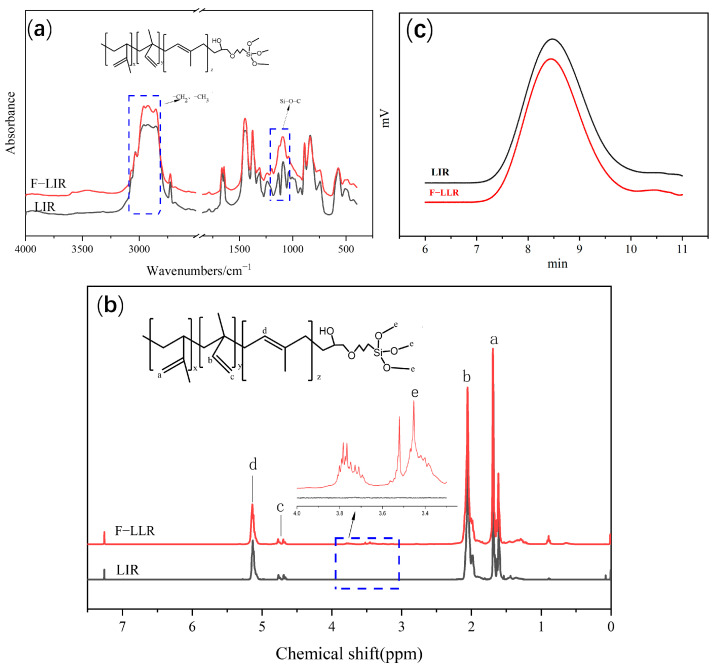
(**a**) The FT-IR spectra of F-LIR and LIR. (**b**) The ^1^H-NMR spectra of LIR and F-LIR. Labeled peaks: a for −CH_3_; b for −CH_2_−; c for =CH_2_, d for −CH=; e for −Si− (O−CH_3_)_3_. (**c**) The GPC elution curve of F-LIR and LIR.

Figure 3a illustrates the FT-IR spectra of F-LIR, LIR and KH-560. The patterns of F-LIR and LIR were very similar. The strong absorbance peaks were observed at 2963 cm^−1^ and 2922 cm^−1^, which were attributed to the C-H stretching vibration of methyl and methylene bonds respectively. The peaks detected at 1663 cm^−1^ was assigned to the stretching vibration of the carbon–carbon double bonds. In addition, the absorbance peaks observed at 887 cm^−1^ and 835 cm^−1^, which were assigned to the out-of-plane bending vibration of the -C=CH_2_ (3,4-structure double bonds) and the -CH=CH_2_ (1,4-structure double bonds), while the spectra of the F-LIR and LIR were different at 1170~970 cm^−1^, which were assigned to the Si-O-C stretching vibration. Compared with the latter, the peak height of the absorption peak observed between 1170 cm^−1^ and 970 cm^−1^ was higher, and the peak area was larger. Furthermore, the characteristic epoxy group absorption peak of KH-560 at 910 cm^−1^ disappeared in the FT-IR spectrum of F-LIR, suggesting that the epoxy group participated in the end-capping reaction during polymerization. These results confirm that alkoxysilane groups were successfully chemically bonded to the chain end of LIR rather than being physically adsorbed species, as they remained stable after repeated washing with n-hexane and ethanol.

The ^1^H-NMR spectra of LIR and F-LIR are shown in Figure 3a,b. The peak was displayed at 4.85~5.15 ppm, which was assigned to the resonance peak in d-CH= of the 1,4-structure. In addition, the resonance peaks indicated at 4.25~4.85 ppm were attributed to the vinyl protons in c=CH_2_ of the 3,4-structure. The resonance peaks were observed at 1.64~1.68 ppm and 1.56~1.62 ppm, which belonged to the methyl protons in a-CH_3_ of the Cis-1,4-structure and Trans-1,4-structure respectively. The ^1^H-NMR spectra of F-LIR and LIR were different at the chemical shift value of e3.4~3.5 ppm, which pertained to the methyl protons in -Si-(O-CH_3_)_3_. Compared with the latter, an absorption peak appeared at 3.5 ppm after end-modified LIR, visualizing successful attaching of silicon–methoxy groups to the end of the LIR. According to the peak area values of these microstructures, the 1,4-strcture and the 3,4-structure amounts of polymer and the end-functionalization efficiency are 93.72%, 6.28% and 59.95% respectively. The end-functionalization efficiency is strongly influenced by the conditions of the capping reaction, and a higher capping efficiency is generally desirable. However, due to the unavoidable influence of impurities and side reactions in the polymerization system, it is almost impossible to achieve a 100% end-functionalization efficiency. Furthermore, no characteristic resonance corresponding to the 1,2-structure was detected at approximately 5.70 ppm, indicating that the polymer predominantly consisted of 1,4- and 3,4-configurations. These data indicated that after the addition of the functionalization agents, the microstructure of the main chains did not change, but only the additive reaction occurred at the end of the anion active species. The combined FT-IR and ^1^H-NMR results confirmed the successful end-functionalization of LIR by KH-560.

The GPC elution curves of F-LIR and LIR are shown in Figure 3c. Both were independent single peaks, indicating that the synthesized F-LIR and LIR were homopolymers and did not produce other impurities such as low-molecular-weight polymers. At the same time, compared with the LIR, the elution curve of the F-LIR moved forward slightly. In other words, the elution time of the F-LIR (Mn:9200 g·mol^–1^, PDI:2.16) was shorter, which was because the presence of trimethoxy groups at the end of the LIR (Mn:9000 g·mol^–1^, PDI:2.14) would increase the hydrodynamic volume of the polymer. The phenomenon could also indicate the successful grafting of KH-560 at the end of LIR.

### 3.2. Processability

#### 3.2.1. Mooney Viscosity

**Figure 4:**
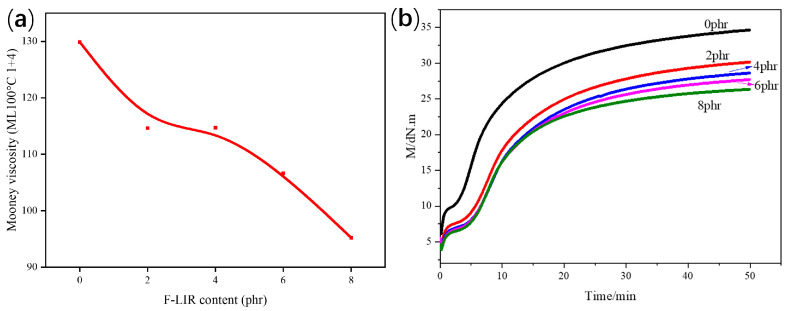
(**a**) Effect of F-LIR content on the Mooney viscosity (ML 1 + 4 at 100 °C) of SSBR/silica compounds. (**b**) Curing curves of SSBR/silica compounds with different F-LIR contents at 160 °C (test time: 50 min).

Mooney viscosity is a common test method which can directly reflect the processing characteristics of rubber compounds. It is well known that the lower the Mooney viscosity value, the better the processability and reduce the damage of the equipment. As shown in Figure 4a, the addition of F-LIR decreased the Mooney viscosity of the SSBR/silica compounds, indicating that, despite the terminal alkoxysilane functionalization, F-LIR effectively plasticizes the tire tread rubber. Notably, when 4 phr of F-LIR was added, the bound rubber content was higher than that of the mixture with 2 phr F-LIR, which helped maintain the Mooney viscosity at a relatively stable level.

#### 3.2.2. Vulcanizing Properties

Figure 4b shows the overall curing curves of silica-filled SBR blends at 160 °C with a test time of 50 min. Parameters including scorch time (t_10_), optimum cure time (t_90_), maximum torque (MH), and minimum torque (ML) are summarized in Table 2. It is observed that the addition of F-LIR could reduce the ML of the blends. Meanwhile, with increasing F-LIR content, the ML of the compound’s values decreased gradually, probably attributing to the plasticizing effect of F-LIR, which was still a small molecular polymer with plasticizing function, although it had the methoxy groups at the end. The MH values also conformed to the above law, indicating that the plasticizing effect of the F-LIR played a leading role. It was known that t_10_, related to the safety of processing, was the delayed action time before the beginning of vulcanization. The higher the t_10_ values, the safer the processing, indicating that the addition of 4 phr and 6 phr F-LIR were the optimal amounts for the process safety. From the cost point of view, 4 phr F-LIR was the optimal content. In addition, the vulcanization rate of the blends decreased with increasing F-LIR content. This behavior may be attributed to the participation of F-LIR in the vulcanization process. Since F-LIR contains unsaturated carbon chains and behaves as a reactive rubber component, the total amount of rubber phase increased with increasing F-LIR loading. In this study, the sulfur content in the formulation was kept constant; therefore, relative to the total rubber content, the effective sulfur concentration decreased slightly as the F-LIR content increased. Furthermore, F-LIR could not only participate in co-crosslinking with the SBR main chains, but also undergo self-crosslinking reactions, which additionally influenced the curing behavior and contributed to a lower curing rate at higher F-LIR loadings.

### 3.3. Dispersion Between SiO_2_-g-F-LIR and SSBR Matrix

The dispersion of filler in rubber matrix was determined by the interaction between filler and polymer. There were three kinds of interaction forces involved in the rubber system, which were the filler–filler networks, filler–polymer interactions and crosslinked network respectively. The homogeneous dispersion of the filler in compounds played a significant role in improving the performance of rubber products.

#### 3.3.1. Payne Effect

**Figure 5:**
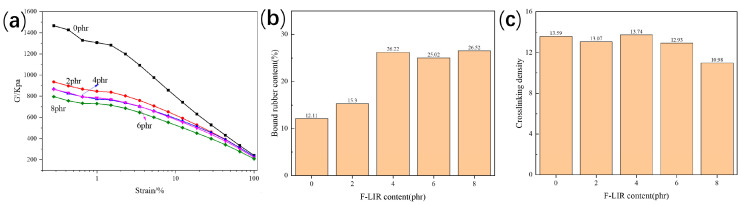
(**a**) Strain-dependent storage modulus (G′) of SSBR/silica composites containing different amounts of F-LIR at 40 °C and 10 Hz. (**b**) Bound rubber content of uncured SSBR/silica composites. (**c**) Crosslink density of SSBR/silica composites determined by equilibrium swelling.

Figure 5a shows the strain-dependent storage modulus (G’) of the blends containing different amounts of F-LIR, which reflects the filler dispersion state in the rubber matrix. When the strain amplitude gradually increased to a critical value, the storage modulus (G’) decreased rapidly; this phenomenon is referred to as the Payne effect. Both filler–filler interactions and filler–rubber interactions contribute to the storage modulus (G’). In generally, the filler–filler network played a dominant role at low strain. Therefore, the pronounced decrease in G’ at low strain is mainly attributed to the breakdown of the filler network structure. A lower Payne effect indicates weaker filler–filler interactions, stronger filler–rubber interactions, and improved filler dispersion. It is observed that the composites without F-LIR had the highest initial G’ and ΔG’ values due to the filler agglomeration, due to severe silica agglomeration and the formation of filler network structures. While the staring G’ and ΔG’ values of the blends with 2 phr F-LIR were lower than the composites without F-LIR. In other words, the G’ and ΔG’ values of the composites with F-LIR were less strain dependence compared with those composites without F-LIR. This result indicates that the terminal alkoxysilane groups of F-LIR reacted with silanol groups on the silica surface, thereby suppressing silica self-aggregation and enhancing filler–rubber interfacial interactions. Moreover, with increasing F-LIR content, the storage modulus curves became progressively flatter. This behavior can be attributed to the combined effects of enhanced silanization and the plasticizing effect of F-LIR. The increased concentration of alkoxysilane groups promoted silica dispersion, while the low-molecular-weight F-LIR improved chain mobility, jointly leading to reduced filler networking and a lower Payne effect.

#### 3.3.2. Bound Rubber Content

To further evaluate the effect of F-LIR on filler–rubber interactions, the bound rubber content was measured, and the results are shown in Figure 5b. Compared with the composite without F-LIR, the incorporation of F-LIR significantly increased the bound rubber content, indicating enhanced interfacial interactions between silica and the rubber matrix. Moreover, the bound rubber content increased progressively with increasing F-LIR loading. This behavior can be attributed to the increased number of alkoxysilane groups capable of reacting with silanol groups on the silica surface, confirming that end-group functionalization is an effective strategy for improving filler–rubber interactions. However, when the F-LIR loading exceeded 4 phr, the increase in bound rubber content became less pronounced, suggesting that the interfacial interaction gradually approached saturation. Combined with the Payne effect results, this phenomenon indicates that the reinforcing effect originating from silanization dominated at low F-LIR loadings (0~4 phr), whereas the plasticizing effect became increasingly significant at higher F-LIR contents.

#### 3.3.3. The Crosslink Density

The crosslink density, determined by equilibrium swelling measurements, reflects the overall network structure and interfacial interactions of the vulcanizates. The results are shown in Figure 5c. The vulcanizate without F-LIR exhibited a relatively high apparent crosslink density, which may be attributed to the dense filler network formed by silica agglomeration, restricting solvent penetration during swelling. After incorporating F-LIR, the crosslinking density slightly decreased when the amount of F-LIR was 2 phr. When the amount of F-LIR was 4 phr, the crosslinking density increased to the highest level. This behavior is attributed to the enhanced interfacial interactions between silica and the rubber matrix as well as the co-crosslinking reaction between F-LIR and SSBR chains, which contributed to the formation of a more effective network structure. However, when the F-LIR loading exceeded 4 phr, the crosslink density gradually decreased. This reduction can be explained by several factors. First, excessive F-LIR consumed sulfur through self-crosslinking reactions, thereby reducing the effective sulfur concentration available for the SSBR network. Second, the low-molecular-weight F-LIR partially contributed to plasticization, which weakened the overall network rigidity. In addition, residual silanol groups on silica could adsorb ZnO and hinder accelerator activity, further suppressing the vulcanization efficiency. Overall, the results suggest that a balance between interfacial reinforcement and plasticization existed in the system, and the optimum network structure was achieved at approximately 4 phr F-LIR.

### 3.4. Mechanical Properties

**Figure 6:**
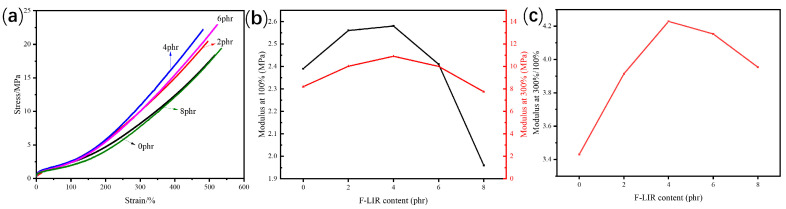
Mechanical properties of SSBR/silica composites with different F-LIR contents: (**a**) stress–strain curves; (**b**) modulus at 100% and 300% elongation; and (**c**) the ratio of modulus at 300% to modulus at 100% elongation.

The mechanical properties of the vulcanizates are shown in Figure 6 and Table 3. The Shore A hardness exhibited a slight variation with increasing F-LIR loading. When the content of F-LIR is 2 and 4 phr, the Shore A hardness value is relatively high, reaching 66 Shore A. This behavior can be attributed to the combined effects of improved silica dispersion and the plasticizing effect of the low-molecular-weight F-LIR, both of which enhanced chain mobility and reduced filler networking. The vulcanizate without F-LIR exhibited greater hardness due to severe silica aggregation and the formation of a rigid filler network structure. In contrast, the incorporation of F-LIR weakened filler–filler interactions and improved interfacial compatibility. However, excessive F-LIR loading (>4 phr) led to a dominant plasticization effect and reduced effective sulfur concentration, which limited the extent of crosslink formation between F-LIR and the SSBR matrix. Consequently, network rigidity decreased, resulting in lower hardness values.

It is well established that improved silica dispersion and stronger filler–polymer interactions positively affect the mechanical properties of vulcanizates, such as tensile strength and modulus at 100% and 300% elongation. The SSBR vulcanizates containing 6 phr F-LIR exhibited the highest tensile strength and stress at given elongation, indicating that this loading facilitated silica dispersion, reduced filler–filler networking, and enhanced interfacial interactions. With increasing F-LIR content, tensile strength initially increased and reached a maximum at 6 phr, then decreased at higher loadings. At low F-LIR loadings (0–6 phr), the improved tensile strength can be attributed to stronger polymer–filler interactions: F-LIR participated in co-crosslinking with SSBR chains and reacted with surface silanol groups on silica, enhancing crosslink density and suppressing filler flocculation. At higher F-LIR contents (>4 phr), the combined effects of self-crosslinking and plasticization contributed to stress concentration and reduced effective sulfur content, leading to a decrease in tensile strength. The 300% modulus and the 100% modulus showed a trend consistent with tensile strength, and reached a maximum at 4 phr. The 300% modulus reflects the crosslinking density of vulcanized rubber. The ratio of 300% to 100% modulus, an indicator of polymer–filler interfacial strength, was higher for F-LIR-containing vulcanizates than for those without F-LIR. Among all samples, the 4 phr F-LIR vulcanizates exhibited the highest ratio, demonstrating the strongest interfacial interaction. In summary, the addition of F-LIR improved filler dispersion and enhanced polymer–filler interactions, thereby significantly improving the mechanical properties of vulcanizates. The optimal performance was observed at 4 phr F-LIR, where 100% and 300% modulus, crosslink density, and bound rubber content reached their maximum values.

### 3.5. Dynamic Mechanical Properties

**Figure 7:**
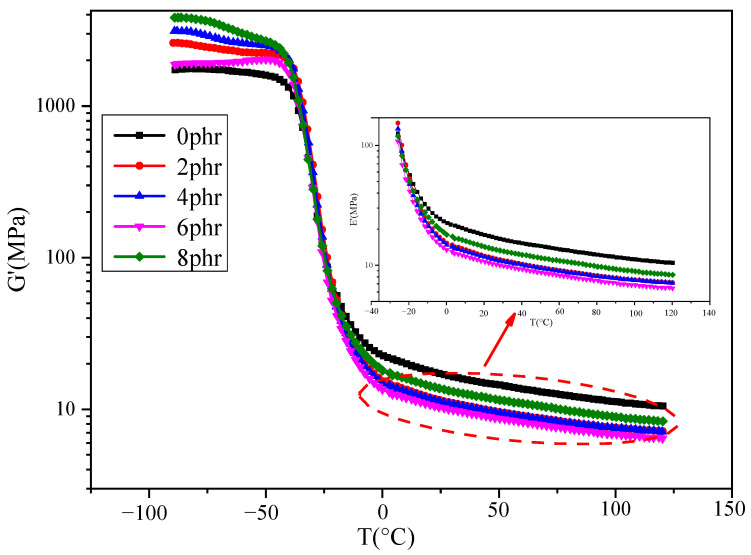
Temperature dependence of the storage modulus (G′) of SSBR/silica vulcanizates with different F-LIR contents.

The temperature dependence of the loss factor tan δ and the storage modulus G’ for all the silica/SSBR vulcanizates under dynamic loading conditions is shown in Figure 7. The storage modulus in the rubbery region is closely related to filler dispersion and filler–rubber interfacial interactions. Strong filler–filler networks formed by silica aggregation can immobilize surrounding rubber chains, thereby restricting chain mobility and increasing the storage modulus. The vulcanizates containing F-LIR exhibited lower G′ values in the rubbery region than the vulcanizate without F-LIR, indicating that the incorporation of F-LIR improved silica dispersion and weakened the filler network structure. This behavior can be attributed to the reaction between the terminal alkoxysilane groups of F-LIR and silanol groups on the silica surface, which suppressed silica aggregation and enhanced filler–rubber interfacial interactions. Moreover, the G′ values of the vulcanizates containing 6 phr F-LIR were significantly lower than those of the other samples, suggesting that filler dispersion and interfacial interactions were substantially improved at these loadings.

**Figure 8:**
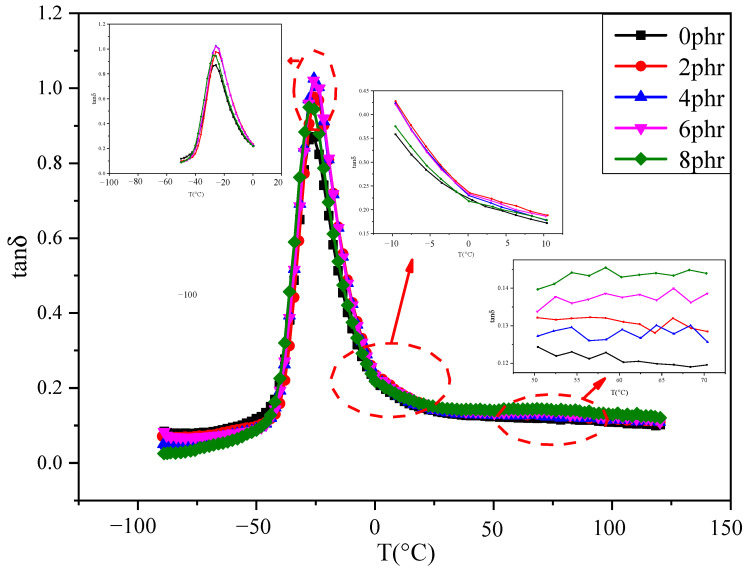
Temperature dependence of the loss factor (tan δ) of SSBR/silica vulcanizates with different F-LIR contents.

The hysteresis behavior of vulcanizates provides important information regarding filler dispersion and interfacial interactions in tread rubber composites. In the glass-transition region, segmental motion of polymer chains is activated. Severe silica aggregation increases the fraction of immobilized rubber chains, thereby reducing the amount of polymer participating in segmental relaxation and leading to a lower tan δ peak intensity. Therefore, stronger filler networking generally results in a lower tan δ peak. As shown in Figure 8, the vulcanizates containing F-LIR exhibited higher tan δ peaks than the vulcanizate without F-LIR, indicating weaker filler networking and improved silica dispersion. This behavior can be attributed to the reaction between the terminal alkoxysilane groups of F-LIR and silanol groups on the silica surface, which suppressed silica aggregation and enhanced filler–rubber interfacial interactions. Among all samples, the vulcanizates containing 4 phr and 6 phr F-LIR exhibited the higher tan δ peak, suggesting the most homogeneous filler dispersion and the strongest interfacial interaction. Combined with the RPA, bound rubber, and crosslink density results, these findings indicate that interfacial reinforcement dominated at low F-LIR loadings (0–4 phr), whereas excessive F-LIR loading led to increased plasticization and reduced effective crosslinking efficiency.

The tanδ values at 0 °C and 60 °C are closely related to the wet skid resistance and rolling resistance of tire tread compounds, respectively. Higher tan δ values at 0 °C are favorable for improved wet skid resistance, whereas lower tan δ values at 60 °C correspond to lower rolling resistance. The vulcanizates containing F-LIR exhibited higher tan δ values at 0 °C than the vulcanizate without F-LIR, suggesting improved wet skid resistance. Moreover, the tan δ values at 0 °C were comparable for the samples containing 2, 4, and 6 phr F-LIR. However, the vulcanizate containing 4 phr F-LIR exhibited the lowest tan δ value at 60 °C, indicating the lowest rolling resistance among all F-LIR-containing samples. Overall, the incorporation of 4 phr F-LIR effectively improved silica dispersion, weakened filler networking, and enhanced filler–rubber interfacial interactions, thereby achieving an improved balance between wet skid resistance and rolling resistance.

## 4. Conclusions

Functionalized liquid polyisoprene rubber (F-LIR)-containing terminal alkoxysilane groups was successfully synthesized through end-group functionalization during anionic polymerization. FT-IR, ^1^H-NMR, and GPC analyses confirmed the successful introduction of silyl groups onto the chain ends of LIR without altering the microstructure of the polymer backbone, and an efficiency of ~60% was achieved. Incorporation of F-LIR enhanced silica dispersion and filler–rubber interfacial interactions in SSBR composites. The reactive F-LIR not only acted as a plasticizer but also participated in co-crosslinking with the matrix, reducing the Payne effect and improving bound rubber content, curing behavior, and mechanical properties. Vulcanizates containing 4 phr F-LIR exhibited the best overall performance, with optimal tensile strength, modulus, and interfacial reinforcement while maintaining a favorable balance between wet skid and rolling resistance. These results demonstrate that F-LIR is an effective multifunctional additive and a promising strategy for high-performance green tire materials.

## Figures and Tables

**Table 1:** Formulation of SSBR/silica compounds (phr).

Ingredient	Loading/phr ^a^
SSBR-2525	100
silica	50
F-LIR	0, 2, 4, 6, 8
Zinc oxide	3
Stearic acid	1
RD ^b^	1
Sulfur	2
TBBS ^c^	1.5
Accelerator D ^d^	1

Note: ^a^ Parts-per-hundred rubber; ^b^ Poly(1,2-dihydro-2,2,4-trimethyl-quinoline); ^c^ N-tert-butylbenzothiazole-2-sulphenamide; ^d^ 1,3-Diphenylguanidine.

**Table 2:** Vulcanization properties of SSBR/silica compounds with various F-LIR loadings at 160 °C.

Title 1	0 phr	2 phr	4 phr	6 phr	8 phr
T10 (min)	0.83	1.29	1.7	1.77	1.53
T90 (min)	26.63	28.93	29.08	28.49	26.46
ML	6.27	4.51	4.23	4.09	3.91
MH	34.64	30.16	28.62	27.71	26.35
MH-ML	28.37	25.65	24.39	23.62	22.44

**Table 3:** Mechanical properties of SSBR/silica composites with different F-LIR contents.

Title 1	0 phr	2 phr	4 phr	6 phr	8 phr
Tensile strength (MPa)	18.43	20.45	22.22	22.92	19.38
Modulus at 100% (MPa)	2.39	2.56	2.58	2.41	1.96
Modulus at 300% (MPa)	8.20	10.02	10.91	10.01	7.75
Elongation at break (%)	517	496	481	523	534
Tear strength (KN/m)	34.88	35.40	36.07	36.54	32.43
Shore A hardness	65	66	66	65	64

## Data Availability

The original contributions presented in this study are included in the article. Further inquiries can be directed to the corresponding author.

## Abbreviations

The following abbreviations are used in this manuscript:

F-LIR	Functionalized liquid polyisoprene rubber
SSBR	Solution styrene–butadiene rubber
TMS	Tetramethylsilane
GPC	Gel permeation chromatography
RPA	Rubber process analyzer
DMA	Dynamic mechanical thermal analyzer
